# Linking hypoxia-inducible factor (HIF)-1α and HIF-2α expression in head and neck cancer

**DOI:** 10.6026/973206300210703

**Published:** 2025-04-30

**Authors:** Pratiksha Kumar, Megha Prabhakar, Shilpa Motghare, Arpita Srivastava, Karandeep Singh, Meena Jain, Megha Patel

**Affiliations:** 1 Department of Oral Pathology and Microbiology, Government College of Dentistry Indore, Madhya Pradesh, India; 2 Department of ENT, SRVS Medical College Shivpuri, Madhya Pradesh, India; 3 Department of Pathology, SRVS, Government Medical College, Shivpuri, Madhya Pradesh, India; 4 Department of Oral Medicine and Radiology, Government College of Dentistry Indore, Madhya Pradesh, India; 5 Department of Oral & Maxillofacial Surgery, SGT University, Budhera Village, Gurugram, Haryana-122505, India; 6 Department of Public Health Dentistry, Santosh Dental College, Santosh Deemed to be University, Ghaziabad, Uttar Pradesh, India; 7 Department of Pediatric and Preventive Dentistry, Karnavati School of Dentistry, Karnavati University, Gandhinagar, Gujarat, India

**Keywords:** Hypoxia-inducible factor, HIF-1α, HIF-2α, head and neck cancer, prognosis, biomarker, immunohistochemistry

## Abstract

Hypoxic conditions often occur in solid tumors like those of the head and neck area, thus enhancing tumor evolution while causing both therapeutic resistance and worsening clinical outcomes. Therefore, it is of interest to evaluate the expression of HIF-1α and HIF-2α proteins to correlate with clinical outcomes in patients with head and neck squamous cell carcinoma (HNSCC). Hence, 80 patients with HNSCC participated in this study. The laboratory analysis of tumor tissue, conducted through immunohistochemistry, revealed the expression levels of HIF-1α and HIF-2α. The examined tissues were categorized into low, moderate, and high expression categories based on immunoreactivity scoring. The clinical outcomes of head and neck cancer patients become worse when producers of HIF-1α and HIF-2α elevate in their tumors.

## Background:

The worldwide medical concern regarding Head and Neck Squamous Cell Carcinoma (HNSCC) persists because this condition ranks sixth in cancer cases, and doctors diagnose about 600,000 new cases yearly [1]. The survival rates for advanced-stage HNSCC have remained minimally improved over the past few decades despite advancements in surgical radiotherapy and chemotherapeutic treatments [2]. The scientific community has increased its recognition of tumor hypoxia as a key variable for cancer development as well as treatment failure alongside diminished clinical results [3]. Hypoxic conditions trigger the activation of hypoxia-inducible factors (HIFs), which regulate transcription factors that control genetic expression, specifically in angiogenesis, as well as metabolism, proliferation, and survival pathways [4].

The research community primarily focuses on studies of HIF-1α and HIF-2α among all HIF isoforms. The subunits maintain stability in hypoxic environments before entering the nucleus, where they pair up with HIF-1β to initiate hypoxia-responsive gene transcription [5]. Although HIF-1α exhibits cancer-aggressive effects and impairs therapy response in various cancers, including HNSCC [6], research indicates that HIF-2α plays essential roles with distinct phenotypic characteristics in tumor development [7]. The clinical value of evaluating HIF-1α and HIF-2α expression in diagnosing head and neck cancer yields conflicting results between research studies, possibly due to variations in tumors and the methods used to detect these proteins [8]. The research has revealed HIF-mediated signaling pathways, along with cellular mechanisms such as epithelial-mesenchymal transition (EMT), ferroptosis resistance, and immune evasion, as these factors drive tumor aggressiveness and treatment resistance in HNSCC [9, 10]. The resistance of tumor cells to ferroptosis develops when HIF-1α stimulates lactate production and enhances amino acid transporter activity, thereby supporting cancer cell survival in hypoxic environments [11]. Tumor invasiveness, along with metastatic potential, gets enhanced by HIF-2α through its interactions with Notch and EGFR pathways, thus demonstrating significant roles in cancer development [12, 13].

The role of HIFs in head and neck cancer prognosis requires additional study as research continues to measure their exact clinical significance. Most systematic reviews and meta-analyses yield mixed findings regarding HIF overexpression and prognosis, as some studies demonstrate a clear association, while others reveal no statistically significant link between these [14, 15]. Prospective research utilizing standardized immunohistochemical techniques for HIF, along with comprehensive clinical follow-up, is essential. The present study examines the expression levels of both HIF isoforms across HNSCC patient samples, comparing their expression data with survival and clinical characteristics. Therefore, it is of interest to study HIF-1α and HIF-2α expression levels in HNSCC tissues, as well as their impact on patient outcomes for gleaning tumor specific biological information to develop novel markers and therapeutic targets.

## Materials and Methods:

## Study design and participants:

The researchers conducted a prospective observational study at an oncology center over an 18-month period. The researchers included 80 patients diagnosed through histological examination of head and neck squamous cell carcinoma (HNSCC). Patients diagnosed with newly diagnosed and previously untreatable HNSCC comprised the selected group for this study. The study excluded patients who received prior chemotherapy or radiotherapy treatment and those with recurrent tumors, as well as patients suffering from systemic conditions that impact survival rates.

## Tissue sampling and processing:

A diagnostic biopsy procedure offered Formalin-fixed, paraffin-embedded tumor tissue, which was used for the study. Researchers cut 4-micrometer-thick slides from paraffin-embedded tissue before placing them onto glass slides coated with poly-L-lysine for immuno-histo-chemical testing.

## Immunohistochemistry (IHC):

The evaluation of HIF-1α and HIF-2α expression was performed using standard peroxidase complex applications between streptavidin and biotin. The prepared slides underwent deparaffinization and rehydration, as well as citrate buffer (pH 6.0) antigen retrieval in a pressure cooker system. The experimental material underwent a process involving a peroxide solution that blocked endogenous peroxidase activity. The tissue sections required overnight incubation at 4°C using primary monoclonal HIF-1α and HIF-2α antibodies at a 1:100 dilution. The research continued with the use of secondary antibodies, followed by the application of chromogen (DAB). The analysts counterstained slides with hematoxylin before affixing them to glass.

## Scoring of immuno-reactivity:

Pathologists who were not familiar with the clinical data independently evaluated the staining intensity levels and the percentages of tumor cells positive for the proteins. The immunoreactive score calculation involved multiplying the level of positive cells (ranging from 0 to 4) by the staining intensity (ranging from 0 to 3). The scores fell into three groups, where low indicated values between zero and four, moderate covered five to eight points, and high equaled scores from nine to twelve.

## Follow-up:

The clinical evaluation took place after outcome assessment every three months for two years for every patient. Patients survived until they passed away for any reason during the observation period, which we defined as overall survival (OS). The measurement of disease-free survival began at the moment of the first treatment and ended at the occurrence of disease recurrence or metastasis.

## Statistical analysis:

Statistical analysis was performed using SPSS version 25.0. The researchers presented categorical variables in terms of frequencies and percentages, while mean values with corresponding standard deviation ranges represented continuous variables. The researchers conducted a comparison between groups by utilizing the chi-square test. Survey analysis utilized Kaplan-Meier curves for inspecting survival data, and the log-rank test was conducted to investigate differences between survival curve patterns. A Cox proportional hazards analysis determined independent prognostic factors. The research determined statistical significance through a p-value cut-off point of 0.05.

## Results:

This research study enrolled 80 patients whose disease diagnosis showed confirmed head and neck squamous cell carcinoma. The study participants had an average age of 56.4 years and a standard deviation of 10.2 years, with a male-to-female ratio of 3.2 to 1. Forty percent of tumors occurred in the oral cavity, while 28.8% originated from the oropharynx and 21.2% from the larynx. Stage III and IV represented the diagnostic conditions in 62.5% of all patients at the time of their initial diagnosis. The research included detailed information on demographic and clinical aspects, which is presented in Table 1.

Immunohistochemical analysis revealed that 45 patients (56.3%) displayed elevated HIF-1α expression, 20 patients (25%) exhibited medium expression levels, and 15 patients (18.7%) showed low HIF-1α expression. The expression results for HIF-2α showed that 47.5% of patients had high levels, 31.3% had moderate levels, and 21.2% exhibited low expression. The analysis revealed dual high expression of these markers in 28 patients (35%). Results indicated that tumor patients presented with advanced cancer stage and lymph node involvement as well as poor differentiation when showing elevated HIF-1α expression (p=0.014, p=0.032, p=0.021). The results revealed that HIF-2α overexpression was associated with an advanced clinical stage (p = 0.011) and lymphovascular invasion (p = 0.038), as shown in Table 2.

The research period of 24 months revealed that 34 patients suffered disease recurrence while 29 patients passed away. The 2-year overall survival rates showed a significant difference between patients with high HIF-1α expression (38%) and those with low or moderate expression (72%) (p = 0.002), according to Kaplan-Meier survival analysis. An elevated expression of HIF-2α was associated with lower disease-free survival rates among patients, with 34% versus 68% (p = 0.006). The independent risk factors that predicted poor outcomes were HIF-1α and HIF-2α based on data from Multivariate Cox regression analysis with HR values (2.14 and 1.89) and their respective confidence intervals (95% CI=1.21-3.76 and 95% CI=1.08-3.29) and p values (0.008 and 0.015).

## Discussion:

The research examined the expression of hypoxia-inducible factor HIF-1α and HIF-2α in head and neck squamous cell carcinoma tissue samples to analyze their clinical implications about patient prognostic information. The study results show that the raised expression of HIF-1α, together with HIF-2α, directly correlates with more severe disease stages and lymph node metastasis, while having negative impacts on patients' survival rates, thus indicating potential use as prognostic biomarkers. Hypoxia is a primary feature of tumor microenvironments, playing key roles in the evolution of solid malignancy diseases and therapeutic resistance in HNSCC, as well as in other solid malignancies [1, 2]. The hypoxic response remains regulated through the protein activities of HIF-1α and HIF-2α, which control genes involved in angiogenesis, metabolism, and apoptosis functions [3, 4]. Previous examinations have revealed that increased HIF-1α levels indicate worse therapeutic outcomes in patients with head and neck cancers, as reported in five and six other studies [5, 6]. The tumor-promoting action of the HIF-1α protein leads to the activation of vascular endothelial growth factor (VEGF), glucose transporter-1 (GLUT1), and hypoxia-related genes [7, 8]. Among study subjects, HIF-1α protein expression levels were high in 56.3% of patients, which correlated with terminal-stage cancer development and worse survival statistics [9, 10]. In hypoxic and nutrient-depleted environments, HIF-2α regulates a distinct set of target genes while acting as a tumorigenic factor [11, 12]. Current research on HNSCC reveals that HIF-2α functions independently to enhance tumor progression and metastasis [13, 14], according to recent findings, despite limited analysis of this protein in HNSCC compared to HIF-1α. Our study found co-expression of HIF-1α and HIF-2α in 35% of the investigated cases, which could indicate their combined role in hypoxia signaling pathways, based on molecular profiling analysis from previous studies [15]. The high expression of HIF-1α and HIF-2α together in patients indicated the worst clinical outcome, as hypoxia-mediated regulation showed synergistic effects [16, 17]. The enzymatic staining methods of immunohistochemistry have created the possibility of determining HIF localization while performing both descriptive and quantitative evaluations. Studies employing various detection antibodies and evaluation scoring systems, along with subjective interpretation differences, result in dissimilar outcomes between research groups [18, 19]. The validation of further findings can be achieved through future research that implements Western blotting alongside RT-PCR. Survival analysis demonstrated that HIF-1α and HIF-2α serve as separate predictors of patient survival, as determined by multivariable Cox regression analyses. The findings from this study show concurrence with research on breast cancer together with lung and renal carcinoma investigations [20, 21-22]. HIF pathway inhibition shows promise as a therapeutic approach, as several inhibitors are currently under development, spanning both preclinical and clinical stages [23]. The study's conclusions could be limited by the narrow sample population collected at a single institution, which reduces the potential external application of the results. The study did not extensively research the molecular systems responsible for HIF-driven tumor evolution.

## Conclusion:

The clinical data indicates that HNSCC patients with elevated HIF-1α and HIF-2α expression experience worse outcomes along with unfavorable pathological characteristics. The identified markers can serve as both predictive indicators for HNSCC outcomes and as treatment targets that will enhance future management approaches.

## Figures and Tables

**Table 1:** Demographic and clinical characteristics of study participants (n=80)

**Variable**	**Frequency (%)**
Age (mean ± SD)	56.4 ± 10.2 years
Gender	
Male	61 (76.3%)
Female	19 (23.7%)
Tumor Site	
Oral cavity	32 (40.0%)
Oropharynx	23 (28.8%)
Larynx	17 (21.2%)
Others	8 (10.0%)
Clinical Stage	
Stage I-II	30 (37.5%)
Stage III-IV	50 (62.5%)

**Table 2:** Correlation of HIF-1α and HIF-2α expression with clinico-pathological features

**Parameter**	**HIF-1α High (n=45)**	**HIF-2α High (n=38)**	**p-value (HIF-1α)**	**p-value (HIF-2α)**
Advanced Stage (III-IV)	37 (82.2%)	33 (86.8%)	0.014*	0.011*
Lymph Node Involvement	30 (66.7%)	28 (73.7%)	0.032*	0.044*
Poor Differentiation	28 (62.2%)	25 (65.8%)	0.021*	0.069
Lymphovascular Invasion	19 (42.2%)	20 (52.6%)	0.058	0.038*
*Statistically significant (p<0.05)
